# Ambient Air Pollution, Meteorological Factors and Outpatient Visits for Eczema in Shanghai, China: A Time-Series Analysis

**DOI:** 10.3390/ijerph13111106

**Published:** 2016-11-08

**Authors:** Qiao Li, Yingying Yang, Renjie Chen, Haidong Kan, Weimin Song, Jianguo Tan, Feng Xu, Jinhua Xu

**Affiliations:** 1 Department of Dermatology, Huashan Hospital, Fudan University, Shanghai 200040, China; 06307070005@fudan.edu.cn; 2 Department of Women’s and Children’s Health Care, Shanghai First Maternity and Infant Hospital, Tongji University School of Medicine, Shanghai 201204, China; yyjenny1214@163.com; 3 Key Laboratory of Public Health Safety of the Ministry of Education and Key Laboratory of Health Technology Assessment of the Ministry of Health, School of Public Health, Fudan University, Shanghai 200040, China; crj_1986@163.com (R.C.); kanh@fudan.edu.cn (H.K.); wmsong@shmu.edu.cn (W.S.); 4 Shanghai Key Laboratory of Meteorological and Health, Shanghai 200135, China; tanjg@mail.typhoon.gov.cn

**Keywords:** eczema, air pollution, meteorological factors, outpatient visit, time-series study

## Abstract

Environmental irritants are important risk factors for skin diseases, but little is known about the influence of environmental factors on eczema incidence. In this time-series study, our objective was to examine the associations of environmental factors with outpatient visits for eczema. Daily outpatient visits between 2007 and 2011 (1826 days) were collected from Huashan Hospital in Shanghai, China. We used an overdispersed generalized additive model to investigate the short-term association between environmental factors and outpatient visits for eczema. Daily outpatient visits for eczema were significantly associated with air pollution and meteorological factors. For example, a 10 μg/m^3^ increase of 7-day (lag 06) average concentrations of PM_10_ (particulate matter no greater than 10 microns), SO_2_, NO_2_ was associated with 0.81% (95% confidence intervals (CI) 0.39%, 1.22%), 2.22% (95% CI: 1.27%, 3.16%) and 2.31% (95% CI: 1.17%, 3.45%) increase in outpatient visits for eczema, respectively. A 10 °C elevation of temperature on lag 0 day were associated with 8.44% (95% CI: 4.66%, 12.22%) increase in eczema visits, whereas 10 unit decrease of 7-day average relative humidity were associated with 10.86% (95% CI: 8.83%, 12.89%) increase in eczema visits. This study provided clear evidence of ambient air pollution, high temperature and low relative humidity on increasing the incidence of eczema in Shanghai, China.

## 1. Introduction

Eczema is a repeated chronic pruritic dermatosis which has the characteristics of fierce itching, dry skin and skin lesions with typical distribution [1]. The prevalence of eczema in childhood has been rising in recent decades, and the risk factors of this have been proposed [2]. For example, the incidence was 2%–3% before 1960, 2%–9% after 1970, and recently has been as high as 20% [3,4]. The causes of the elevation of incidence are still unrevealed.

It was previously reported that, beyond a genetic background [5], eczema was related to factors including food intake, lifestyle and social and economic status, which were notably and positively associated with incidence and pathogenic condition [6]. Other important determinant factors are related to the environment, including interior environmental conditions, the relative degree of humidity and temperature. Moreover, skin symptoms may be induced by daily biological and chemical materials [7]. Ambient air pollution was recognized as a crucial risk factor in various human diseases, mainly including elevated morbidity and mortality from cardiopulmonary diseases [8,9,10,11,12]. Moreover, air pollution may also induce some allergic diseases (e.g., asthma) [13]. Although in vivo and in vitro studies have shown that particulate matters (PM) can be deleterious to skin [14,15,16], few epidemiological investigations have been conducted to evaluate the relationship between polluted atmosphere and skin conditions [17,18]. It has been shown that applying epicutaneous air-borne allergens could induce eczema [19,20]. Furthermore, ambient air pollution was related to elevated risks of allergic diseases, including exacerbating pre-existing asthma and accelerating the development of allergic sensitization or atopic diseases [21,22,23]. Several other studies have examined the associations between air pollutants and atopic dermatitis [23,24], but there were few epidemiologic findings, and the existing evidence was inconsistent.

Inappropriate weather conditions were also important risk factors for human health including allergic and skin diseases. It was reported that some inhaled allergens might induce seasonal allergic rhinitis [25]. Eczema typically exhibits a seasonal variation, so ambient temperature is assumed to play an important role in eczema incidence. It was also reported that humidity might be correlated with eczematous eruptions [25,26,27]. However, the associations between environmental temperature, humidity, and eczema incidence have thus far barely been assessed in a direct way. 

Therefore, in this study, the associations between outdoor air pollution, temperature as well as humidity and eczema incidence were characterized by outpatient visits of a large hospital in Shanghai, China. 

## 2. Materials and Methods

### 2.1. Data

Shanghai lies in the east end of Chang River Delta and has the highest population density in China. Shanghai has a subtropical humid monsoon climate, which shows distinct seasons and sufficient water supply. In summer, its temperature peaks in July and August. Data on hospital outpatient visits for skin conditions between 1 January 2007 and 31 December 2011 (1826 days) were collected from Huashan Hospital Fudan University, which is located in the downtown area of Shanghai. Huashan Hospital has the largest department of dermatology in Shanghai even in China. Daily cases of the outpatient visits for eczema (ICD code: L30.9) were obtained, and patients living outside the urban areas of Shanghai were excluded according to recorded home addresses. 

The average concentrations of three criteria air pollutants including particulate matter no greater than 10 microns (PM_10_), nitrogen dioxide (NO_2_) and sulfur dioxide (SO_2_) in 24 h during the period of this study were obtained from the Shanghai Environment Monitoring Center. The average daily concentration of each pollutant was calculated from the available monitoring data of stations located in six urban districts under the China National Quality Control. While calculating the 24-h average concentrations, at least 75% hourly measurements should be available in that day. The monitoring stations were mandated not to be in the direct vicinity of apparent air pollution sources.

Daily relative humidity and temperature data in the period of investigation were collected from a fixed-site station (Xujiahui) regulated by Shanghai Meteorological Bureau.

### 2.2. Statistical Methods

The time-series method has been widely applied for investigating the acute effects of air pollutants and weather conditions. In this study, the short-term associations between daily eczema outpatient visits and air pollutants as well as weather variables were estimated by an overdispersed generalized additive model (GAM). Because there were no studies showing an obvious nonlinear association between air pollutants, weather conditions, and eczema incidence, we introduced their linear terms one at a time in the models. Moreover, a few covariates were also introduced in GAM, including: (1) a natural cubic smooth function of calendar day (7 degrees of freedom per year) to exclude unmeasured long-term and seasonal trends of outpatient visits; (2) an indicator variable for day of the week; (3) a binary variable for holidays. 

The potentially lagged effects were examined using various lag (L) structures, i.e., single day lag (from L0 to L6) and multiple-day average lag (from L01 to L06). For single-day lag, a lag of zero day (L0) is corresponding to concentration in the present day while a lag of one day (L1) is corresponding to the concentration in previous day. In a model of multiple-day average lag, L0*x* is corresponding to the moving average concentrations of current-day and previous *x* days. For example, L06 corresponds to a seven-day moving average of concentration of the present day and previous six days. The exposure and response correlation curves between air pollution and outpatient visits for eczema were plotted using a spline function in the GAM, and the results figures showed approximately linear shape. To allow for estimate the effects, linear terms were introduced in the basic models.

Moreover, bi-pollutant models were used to evaluate the robustness of our results from the single-pollutant models after adjusting for the simultaneous exposure to other pollutants. In stratification analysis, the modification by season was also tested by dividing the study period into warm season (from April to September) and cool season (from October to March). 

All of the statistical analyses were two-sided, and at a 5% level of significance. All analyses were conducted using R software (version 3.1.2, R Foundation for Statistical Computing, Vienna, Austria [28]) with the GAM fitted by the “mgcv” package. The effect estimates were expressed as the percent changes and their 95% confidence intervals (CIs) in daily outpatient visits for eczema associated with a 10 μg/m^3^ increase in air pollutant concentrations or a unit change in weather conditions.

## 3. Results

Between 1 January 2007 and 31 December 2011 (1826 days), a total of 510,158 eczema visits for skins conditions were recorded at the Department of Dermatology of Huashan Hospital. On average, there were 279 cases of eczema per day, accounting for 19% of the total visits at this department (Table 1). 

Table 1 also summarizes the statistics on air pollutant concentrations and weather conditions during the study period. The annual average concentrations of PM_10_, SO_2_ and NO_2_ were 83 μg/m^3^, 42 μg/m^3^ and 60 μg/m^3^, respectively. The levels of PM_10_ and SO_2_ were much higher than those reported in Western Europe and North America. The annual average temperature and relative humidity were 18 °C and 70%, respectively, reflecting the typical subtropical climate of Shanghai. The three air pollutants were highly correlated with each other, and moderately correlated with temperature and humidity.

Table 2 shows the effect estimates using different lag days. The effects of PM_10_ and NO_2_ on eczema visits decreased but remained statistically significant from lag 0 to lag 3 days, whereas the effects of SO_2_ could last up to 5 days. Then, their effects attenuated substantially and turned out to be non-significant. The estimates using cumulative lags were much higher than those using single-day lags. For example, a 10 μg/m^3^ increase of 7-day average PM_10_, SO_2_, and NO_2_ corresponds to 0.81% (95% CI: 0.39%, 1.22%), 2.22% (95% CI: 1.27%, 3.16%) and 2.31% (95% CI: 1.17%, 3.45%) increase of eczema visits, respectively.

For meteorological factors, we found a positive association between temperature and eczema, which was statistically significant on the concurrent day (lag 0) and some subsequent days (lag 4 and lag 5). For example, 10 °C elevation of temperature on lag0 was associated with an 8.44% (95% CI: 4.66%, 12.22%) increase in eczema visits. The negative correlation between humidity and eczema outpatient visits was stable and significant using all lags we examined, and cumulative lags generated much higher effect estimates than single-day lags. For example, 10 units decrease of relative humidity at lags 0–6 days were associated with 10.86% (95% CI: 8.83%, 12.89%) increase in eczema visits. 

**Figure 1:**
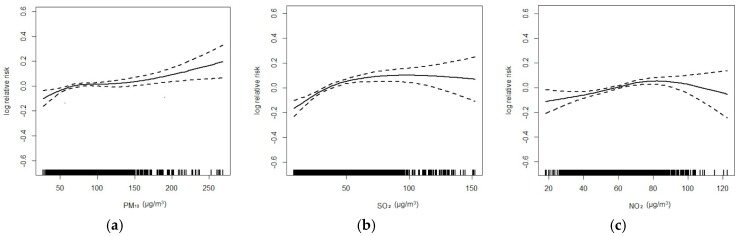
Exposure-response curves for the associations of PM_10_ (**a**); SO_2_ (**b**) and NO_2_ (**c**) with outpatient visits for eczema (lag 06).

**Figure 2:**
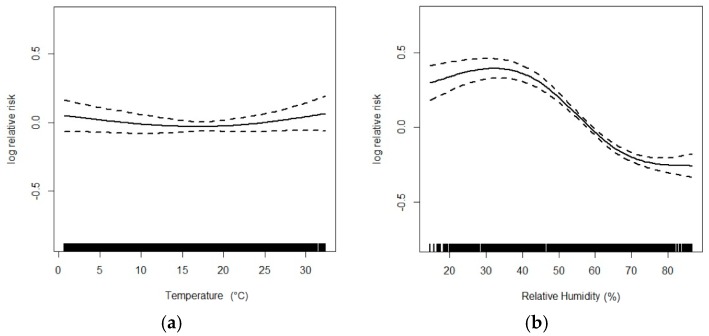
Exposure-response curves for the associations of mean temperature (**a**) and relative humidity (**b**) with outpatient visits for eczema (lag 06).

Figure 1 and Figure 2 flexibly plot the exposure-response relationship curves between various environmental variables and eczema visits. Generally, the curves of three air pollutants were obviously positive but became flat when concentrations were high; the curve was very weak for temperature, but was strongly negative for humidity.

The results of two-pollutant models using lag 06 were shown in Table 3. When adjusting for co-pollutants, the effect estimates of PM_10_, NO_2_ and SO_2_ attenuated a little but were still statistically significant. 

Table 4 presents the effect estimates separated by cool and warm seasons. Generally, effect estimates in warm season were much higher than in the cool season. We only found significant associations of NO_2_ at lag 0 and 1 day. The lag patterns in the warm period were similar to the whole-year period.

## 4. Discussion

It was reported in this investigation that air pollution, high temperature and low humidity were associated with outpatient visits of eczema. Both in the single-pollutant models and two-pollutant models, the effect estimates of PM_10_, SO_2_ and NO_2_ were statistically significant. All of the air pollutants and meteorological factors showed lag effects in the present study. Although the effect size was relatively small per unit change of the exposure variables, our results still have considerable public health significance due to the ubiquitous exposure. Our results contributed to the limited scientific evidence that ambient air pollution and meteorological factors could affect the incidence of eczema.

It was indicated that air pollutants were positively associated with eczema incidence although there were controversies in previous studies. For example, a study involving 1880 students aged 9–13 years did not reveal any association between common air pollutants and onsets of eczema [29], which might be due to limited statistical power associated with the design of the cross-sectional study. The positive association between air pollution and eczema was biologically plausible, though the definite principle was still unrevealed. Air pollutants could exacerbate the dermatological signs of eczema or weaken the protection of skin [30,31,32]. According to recent studies on cell and animal models, a few reasonable mechanisms were proposed. Among these hypotheses, particles could directly induce the production of reactive oxygen species and consequently resulted in oxidative stress, damage and inflammation reactions to the immune system [33]. Exposure to air pollutants including chemicals (toxic metals, etc.) with oxidant-generating capacities may induce neutrophilic inflammation, a decrease in pH, elevated eosinophils and cytokine levels as well as IgE generation [21,34,35].

Previous studies suggested that exposure to air pollutants (NO*_x_*, PM_10_, CO and SO_2_) could induce symptoms of eczema, which are consistent with our findings in most parts [36,37]. A large quantity of individual variables were included in previous studies by means of questionnaires including interior environmental factors, medical histories of parents as well as the social and economic status, which were not available in this retrospective study due to lack of a secondary database. Nevertheless, the correlation between air pollutants exposure and outpatient visits of eczema was strengthened in this study and could be used as the baseline for further investigation. 

Dynamic climate factors might also exert an influence on increasing eczema incidence, but the epidemiological data were still controversial. This time-series investigation discovered a positive correlation between temperature and incidence as well as a strong inverse association between humidity and incidence, which was consistent with most previous studies. It was proposed by a cohort study on children with atopic eczema that skin could be irritated by increased sweat caused by warmth and humidity [38]. This could possibly be used to explain the above phenomenon. It was also proposed by another study that skin dryness, which is a clinical sign of eczema, could be induced by heat and desiccation [39]. For example, in many early case studies as well as observations, low humidity was associated with an eczematous attack [40]. Silverberg reported that eczema incidence was notably lower in the highest-quartile of annual mean relative humidity [40]. Sargen et al. found that high humidity was associated with poorly controlled eczema in children, but this association was not statistically significant in multivariate analysis [39]. It was also evidenced by an international study on climate factors that the humidity and temperature were inversely associated [41]. In contrast, a Spanish study suggested a negative correlation between eczema prevalence and temperature as well as a positive correlation between eczema prevalence and outdoor humidity [42]. Higher relative humidity was biologically protective, possibly because it could improve skin barrier functions which could restrain the cellular pathways related to eczema. The inconsistency in some existing results may be due to differences in location, socio-demographic features and methodologies. Kallawicha, K. et al. reported that wind speed also plays a role in eczema incidence [43]. However, the results were almost same when wind speed was involved in the present study.

The acute influences of air pollutants and climate factors on eczema incidence were investigated based on a large database in Shanghai, China. High air pollution levels and distinct seasons in Shanghai offered sufficient opportunities to explore the effects of environmental risk factors on skin conditions. Nevertheless, our study still had some limitations. On one hand, this time-series study is inherently an ecological analysis, which could not allow for adjustment of individual confounders (such as sex, age, smoking, socio-economic status) or conducting for a modification analyses by medication use and comorbidities. Some studies revealed different health responses to air pollution among different gender and age groups, especially in non-respiratory diseases [44,45,46,47,48], but as a retrospective study, the information of gender and age were not studied in this paper due to limited data accessibility and availability, and total outpatient visit numbers for each day, instead of each patient’s detailed information including age and gender, were obtained. Thus, the data might be to some extent representative of the populated areas of Shanghai. On the other hand, the monitoring results of all stations were averaged and used as the proxy of population exposure level to environmental factors which was widely used in lots of previous time-series studies. This method may raise issues in relation to exposure measurement errors, and subsequently lead to difficulties in interpreting our results, although simulation studies indicated that this kind of measurement error may produce a downward bias [49]. 

## 5. Conclusions

In summary, this study provided clear evidence that ambient air pollution, high temperature, and low relative humidity could increase the incidence of eczema in Shanghai, China. Further studies from both an epidemiological and a physiological perspective are needed to establish the causal relationship between air pollution, meteorological factors, and eczema.

## Figures and Tables

**Table 1:** Summary statistics of outpatient visits for outpatient visits for eczema, air pollutant concentrations and meteorological factors.

Variables	Mean	SD	Minimum	P(25)	Median	P(75)	Maximum
Outpatient Visits for Eczema	279	123	4	198	261	345	772
PM_10_ (μg/m^3^)	83	55	7	48	70	104	600
SO_2_ (μg/m^3^)	42	30	5	20	34	56	261
NO_2_ (μg/m^3^)	60	23	11	43	58	74	174
Temperature (°C)	17	9	−3	10	18	25	36
Humidity (%)	59	18	−1	46	62	73	95

Abbreviations: PM_10_, particulate matter no greater than 10 microns; SO_2_, sulfur dioxide; NO_2_, nitrogen dioxide. SD, standard deviation.

**Table 2:** Percent change (mean and 95% confidence intervals) in daily outpatient visits for eczema associated with 10 unit change of environmental variables using different lag days.

Lags	Mean and 95% CI				
	PM_10_	SO_2_	Temperature	Humidity	NO_2_
0	0.40 (0.18, 0.61)	0.97 (0.40, 1.54)	8.44 (4.66, 12.22)	−2.70 (−3.98, −1.41)	2.15 (1.54, 2.76)
1	0.29 (0.08, 0.50)	0.87 (0.32, 1.41)	3.58 (−0.18, 7.34)	−3.89 (−5.15, −2.64)	1.56 (0.94, 2.17)
2	0.23 (0.01, 0.44)	0.74 (0.20, 1.27)	2.05 (−1.75, 5.85)	−4.06 (−5.28, −2.86)	0.82 (0.21, 1.43)
3	0.25 (0.03, 0.46)	0.87 (0.35, 1.40)	2.30 (−7.18, 11.78)	−4.62 (−5.80, −3.45)	0.68 (0.06, 1.28)
4	0.20 (−0.01, 0.42)	0.96 (0.43, 1.48)	4.84 (1.05, 8.62)	−4.64 (−5.81, −3.46)	0.48 (−0.12, 1.09)
5	0.19 (−0.02, 0.41)	0.84 (0.32, 1.37)	4.14 (0.36, 7.92)	−4.09 (−5.27, −2.91)	0.27 (−0.33, 0.87)
6	0.18 (−0.04, 0.40)	0.51 (−0.01, 1.04)	3.35 (−0.41, 7.11)	−2.88 (−4.06, −1.69)	−0.02 (−0.62, 0.58)
06	0.81 (0.39, 1.22)	2.22 (1.27, 3.16)	2.54 (−3.82, 8.90)	−10.86 (−12.89, −8.83)	2.31 (1.17, 3.45)

Abbreviations: PM_10_, particulate matter no greater than 10 microns; SO_2_, sulfur dioxide; NO_2_, nitrogen dioxide; CI, confidence interval.

**Table 3:** Percent change (mean and 95% CI) in daily outpatient visits for eczema associated with 10 μg/m^3^ change in 7-day moving average air pollutant concentrations using both single-pollutant and two-pollutant models.

Air Pollutants	Model	Estimates, 95% CI
PM_10_	-	0.81 (0.39, 1.22)
	+NO_2_	0.58 (0.16, 0.99)
	+SO_2_	0.71 (0.30, 1.13)
NO_2_	-	2.31 (1.17, 3.45)
	+PM_10_	1.91 (0.73, 3.10)
	+SO_2_	1.99 (0.81, 3.16)
SO_2_	-	2.22 (1.27, 3.16)
	+PM_10_	1.96 (0.99, 2.92)
	+NO_2_	1.51 (0.54, 2.48)

**Table 4:** Percent change (mean and 95% CI) in daily outpatient visits for eczema associated with 10 μg/m^3^ change in air pollutant concentrations, stratified by seasons.

Air Pollutants	Lags	Warm Seasons		Cool Seasons	
PM_10_	0	0.62	(0.38, 0.87)	−0.21	(−0.66, 0.24)
	1	0.56	(0.31, 0.80)	−0.24	(−0.66, 0.18)
	2	0.51	(0.26, 0.75)	−0.33	(−0.65, 0.10)
	3	0.38	(0.13, 0.63)	−0.06	(−0.49, 0.35)
	4	0.16	(−0.09, 0.41)	0.25	(−0.15, 0.65)
	5	0.11	(−0.15, 0.37)	0.32	(−0.09, 0.72)
	6	0.08	(−0.18, 0.35)	0.48	(0.06, 0.89)
	06	1.23	(0.74, 1.73)	0.34	(−0.61, 1.29)
NO_2_	0	2.56	(1.87, 3.24)	1.88	(0.63, 3.14)
	1	1.73	(1.02, 2.43)	1.66	(0.45, 2.88)
	2	1.28	(0.57, 1.98)	0.38	(−0.83, 1.59)
	3	0.99	(0.28, 1.69)	0.21	(−0.98, 1.40)
	4	0.69	(−0.02, 1.41)	−0.06	(−1.26, 1.11)
	5	0.53	(−0.18, 1.24)	−0.43	(−1.62, 0.75)
	6	0.45	(−0.26, 1.15)	−0.83	(−2.02, 0.36)
	06	4.17	(2.78, 5.57)	0.63	(−2.04, 3.29)
SO_2_	0	1.25	(0.52, 1.98)	0.58	(−0.48, 1.63)
	1	1.33	(0.64, 2.03)	0.49	(−0.51, 1.48)
	2	1.37	(0.69, 2.05)	0.12	(−0.87, 1.11)
	3	1.34	(0.66, 2.02)	0.45	(−0.51, 1.40)
	4	1.18	(0.49, 1.87)	0.91	(−0.03, 1.86)
	5	0.95	(0.26, 1.64)	0.90	(−0.03, 1.84)
	6	0.88	(0.19, 1.58)	0.36	(−0.59, 1.32)
	06	3.84	(2.56, 5.11)	2.36	(0.13, 4.58)
